# Computational biology: deep learning

**DOI:** 10.1042/ETLS20160025

**Published:** 2017-11-14

**Authors:** William Jones, Kaur Alasoo, Dmytro Fishman, Leopold Parts

**Affiliations:** 1 Wellcome Trust Sanger Institute, Hinxton, U.K.; 2 Institute of Computer Science, University of Tartu, Tartu, Estonia; 3 Quretec Ltd., Tartu, Estonia

**Keywords:** bioinformatics, computational biology, deep learning

## Abstract

Deep learning is the trendiest tool in a computational biologist's toolbox. This exciting class of methods, based on artificial neural networks, quickly became popular due to its competitive performance in prediction problems. In pioneering early work, applying simple network architectures to abundant data already provided gains over traditional counterparts in functional genomics, image analysis, and medical diagnostics. Now, ideas for constructing and training networks and even off-the-shelf models have been adapted from the rapidly developing machine learning subfield to improve performance in a range of computational biology tasks. Here, we review some of these advances in the last 2 years.

## Introduction

In 2017, it is impossible to avoid the buzz around deep learning. Deep neural networks appear to be a hammer that can crack any nut put in its way, and are thus applied in nearly all areas of research and industry. Originally inspired by models of brain function, neural networks comprise layers of interconnected compute units (neurons), each calculating a simple output function from weighted incoming information (Box 1 and references therein). Given a well-chosen number of neurons and their connectivity pattern, these networks have a seemingly magical ability to learn the features of input that discriminate between classes or capture structure in the data. All that is required is plenty of training examples for learning.

There are two main reasons why deep learning is appealing to computational biologists. First, this powerful class of models can, in principle, approximate nearly any input to output mapping if provided enough data [1]. For example, if the goal is to predict where a transcription factor binds, there is no need to restrict the expressivity of the model to only consider a single sequence motif. Second, deep neural networks can learn directly from raw input data, such as bases of DNA sequence or pixel intensities of a microscopy image. Contrary to the traditional machine learning approaches, this obviates the need for laborious feature crafting and extraction and, in principle, allows using the networks as off-the-shelf black box tools. As large-scale biological data are available from high-throughput assays, and methods for learning the thousands of network parameters have matured, the time is now ripe for taking advantage of these powerful models.

Here, we present the advances in applications of deep learning to computational biology problems in 2016 and in the first quarter of 2017. There are several reviews that broadly cover the content and history of deep learning [2,3], as well as the early applications in various domains of biology [4]. We do not attempt to replicate them here, but rather highlight interesting ideas, and recent notable studies that have applied deep neural networks on genomic, image, and medical data.

## Genomics

The main focus of deep learning applications in computational biology has been functional genomics data. Three pioneering papers [5–7] generalized the traditional position weight matrix model to a convolutional neural network (Box 1, reviewed in ref. [4]), and demonstrated the utility for a range of readouts. All these studies used a multilayer network structure to combine base instances into sequence motifs, and motif instances into more complex signatures, followed by fully connected layers to learn the informative combinations of the signatures.

### New applications to functional genomics data

After demonstrations that deep learning models can outperform traditional approaches in functional genomics, they were widely adopted. Similar convolutional architectures have been applied to predict DNA sequence conservation [8], identify promoters [9] and enhancers [10], detect genetic variants influencing DNA methylation [11], find translation initiation sites [12], map enhancer–promoter interactions [13], and predict transcription factor binding [14]. We present a list of recent studies in the Appendix to this article.

The applications of deep neural networks are not limited to genomic sequences. For example, CODA [15] applies a convolutional neural network to paired noisy and high-quality ChiP-seq datasets to learn a generalizable model that reduces the noise caused by low cell input, low sequencing depth, and low signal-to-noise ratio. Convolutional neural networks have also been used to predict genome-wide locations of transcription start sites from DNA sequence, RNA polymerase binding, nucleosome positioning and transcriptional data [16], as well as gene expression from histone modifications [17], 3D chromatin interactions from DNA sequence and chromatin accessibility [18], DNA methylation from single-cell bisulfite sequencing data [19], and protein binding to RNA from the primary, secondary, and tertiary structures [20] or other features [21].

Fully connected neural networks (Box 1) are often used for standard feature-based classification tasks. In genomics, they have been applied to predict the expression of all genes from a carefully selected subset of landmark genes [22], predict enhancers, [23] and to distinguish active enhancers and promoters from background sequences [24]. An early study also applied an architecture with three hidden layers and 60 neurons to estimate historical effective population size and selection for a genomic segment with reasonable results [25]. However, carefully chosen summary statistics were used as input, so there were limited gains from the traditional benefit of a network being able to figure out relevant features from raw data. While demonstrating good performance, these applications do not make use of the recent advances in neural network methodologies, and we do not describe them further.

### Variant calling from DNA sequencing

With the development of high-throughput sequencing technology, models for the produced data and errors were created in parallel [26,27] and calibrated on huge datasets [28]. Perhaps surprisingly, deep neural networks provided with plenty of data can achieve high accuracies for variant calling without explicitly modeling sources of errors. A four-layer dense network considering only information at the candidate site can achieve reasonable performance [29,30]. Poplin and colleagues further converted the read pileup at a potential variable site into a 221 × 100-pixel RGB image, and then used Inception-v2 [31], a network architecture normally applied in image analysis tasks, to call mutation status [32]. Base identity, base quality, and strand information were encoded in the color channels, and no additional data were used. This approach won one of the categories of the Food and Drug Administration administered variant calling challenge; the authors ascribe its performance to the ability to model complex dependencies between reads that other methods do not account for.

The advantage of deep neural network models also seems to hold for other sequencing modalities. Nanopore sequencing calls convert currents across a membrane with an embedded 5-mer containing pore into bases. One would, thus, expect that a hidden Markov model with four-base memory describes the data adequately, but a recurrent neural network (Box 1) with arbitrary length memory performs even better [33].

Box 1. **Common neural network models**

Neuron, activation function, and neural network

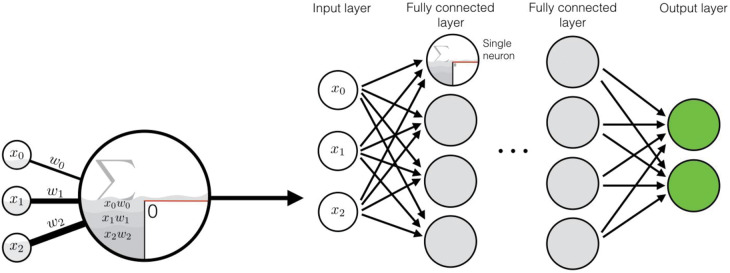

**Synopsis:** A neuron (left) is the basic compute unit of a neural network. Given the values *x*_1_ … *x_N_* of all *N* inputs, it calculates its total input signal by weighting them with the learned weights *w*_1_ … *w_N_*. The total input *w*_1_*x*_1_ + ··· + *w_N_x_N_* is then passed to an activation function [e.g. rectified linear unit, pictured, *y* = max(0, *w*_1_*x*_1_ + ··· + *w_N_x_N_*) or sigmoid, *y* = 1/(1 + exp(−*w*_1_*x*_1_− ··· −*w_N_x_N_*)] that calculates the neuron output, propagated to be the input for the next layer of neurons. In a dense, multilayer network (right), the data are fed as input to the first layer, and the output is recorded from the final layer activations (green).

**Useful for:** general purpose function estimation. Fully connected neurons are often employed in final layer(s) to tune the network to the required task from features calculated in previous layers.

**Classical analogy:** hierarchical models, generalized linear models

**In-depth review:** ref. [2].

Convolutional Neural Networks

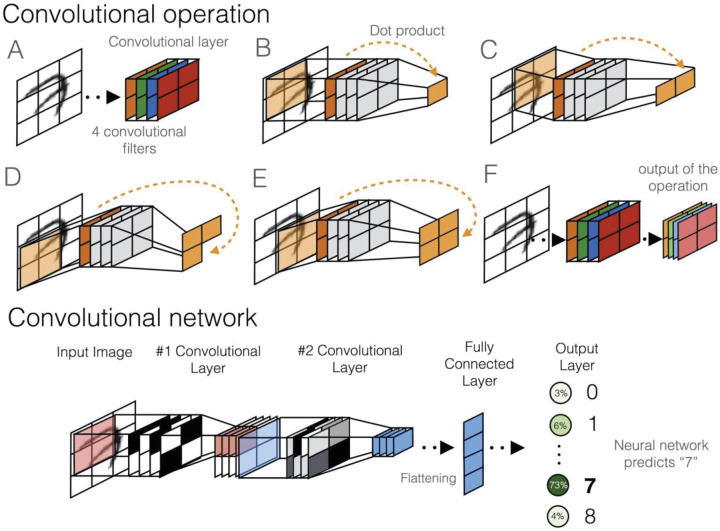

**Synopsis:** These networks harbor special convolutional neurons (‘filters’, different colors in A,F) that are applied one by one to different parts of the input (B–E for four example image parts) with the same weights. This allows the same pattern to be matched regardless of its position in the data (different image patches in example) and therefore reduces the number of parameters that need to be learned. Convolutional networks have one or more layers of convolutional neurons that are typically followed by deeper fully connected layers to produce the output (bottom).

**Useful for:** learning and detecting patterns. Convolutional neurons are usually added in lower-level layers to learn location-independent patterns and pattern combinations from data.

**Classical analogy:** position weight matrix (DNA sequence), Gabor filters (images)

**In-depth review:** ref. [4]

Recurrent Neural Networks

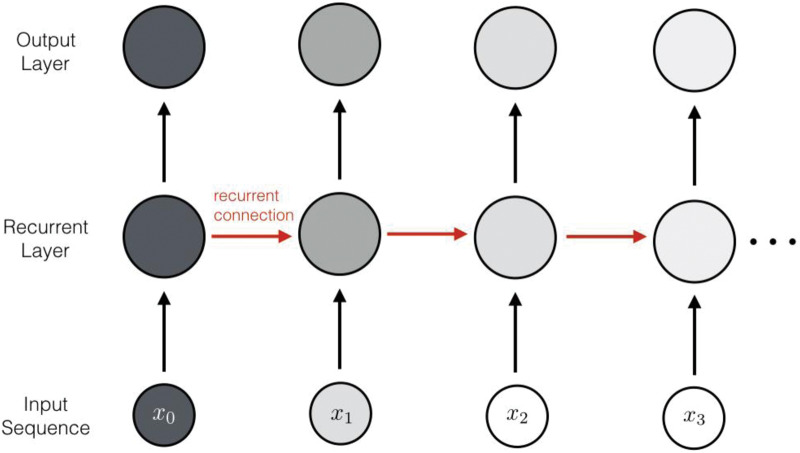

**Synopsis:** Recurrent neural networks typically take sequential data as input (bottom) and harbor connections between neurons that form a cycle. This way, a ‘memory’ can form as an activation state (darkness of neuron) and be retained over the input sequence thanks to its cyclical propagation.

**Useful for:** modeling distant dependencies in sequential data.

**Classical analogy:** Hidden Markov Models

**In-depth review:** ref. [36].

Autoencoders

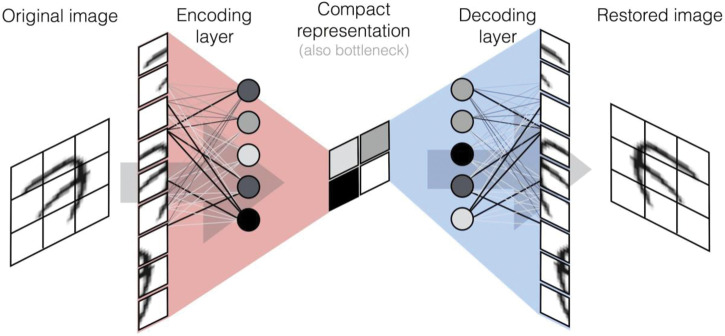

**Synopsis:** Autoencoders are a special case of a neural network, in which input information is compressed into a limited number of neurons in a middle layer, and the target output is the reconstruction of the input itself.

**Useful for:** unsupervised feature extraction

**Classical analogy:** independent components analysis

**In-depth review:** ref. [100].

Generative Adversarial Networks

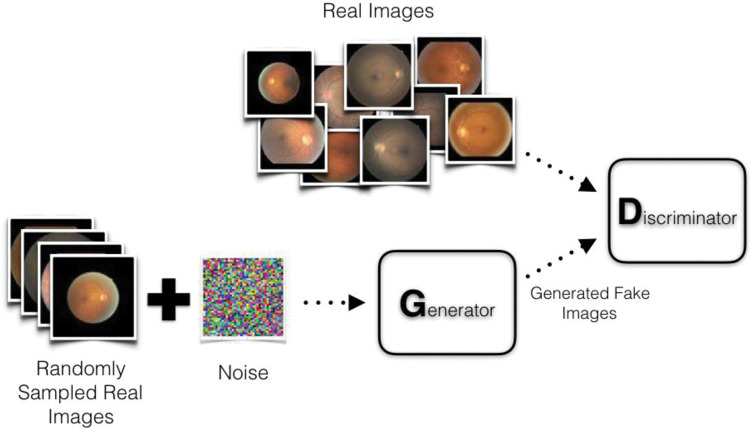

**Synopsis:** a two-part model that trains both a generative model of the data and a discriminative model to distinguish synthetic data from real. The two parts compete against each other, the generator tries to generate images that are passed as real, and the discriminator attempts to correctly classify them as synthetic.

**Useful for:** building a generative model of the data

**Classical analogy:** generative probabilistic models

**Proposing paper:** ref. [81]

### Recent improvements to convolutional models

Building on the successes mentioned above, the basic convolutional model has been improved for accuracy, learning rate, and interpretability by incorporating additional intuition from data and ideas from machine learning literature.

#### Incorporating elements of recurrent neural networks

Three convolutional layers could capture the effects of multiple nearby regulatory elements such as transcription factor binding sites [7]. DanQ [34] replaced the second and third convolutional layers with a recurrent neural network (Box 1), leading to a better performance. In principle, using a recurrent neural network allows extracting information from sequences of arbitrary length, thus better accounting for long-range dependencies in the data. While the DanQ model consisted of convolutional, pooling, recurrent, and dense layers, DeeperBind [35] omitted the pooling layers, thus allowing them to retain complete positional information in the intermediate layers. SPEID [13] further proposed an elegant way to modify the DanQ network by taking sequence pairs, rather than single-DNA sequences, as input, to predict enhancer–promoter interactions. In an interesting application, DeepCpG [19] combined a nucleotide-level convolutional neural network with a bidirectional recurrent neural network to predict binary DNA methylation states from single-cell bisulfite sequencing data. An important caveat to the general applicability of recurrent neural networks is that they can be difficult to train, even with the recent improvements in methodology [8,36].

#### Reverse complement parameter sharing

Shrikumar et al*.* [37] noted that convolutional networks for DNA learn separate representations for the forward and reverse complement sequences. This led to more complicated and less stable models that sometimes produced different predictions from the two strands of the same sequence. To overcome these limitations, they implemented new convolutional layers that explicitly share parameters between the forward and reverse complement strands. This improved model accuracy, increased learning rate, and led to a more interpretable internal motif representation.

#### Incorporating prior information

A key advantage of neural networks is that, given sufficient data, they learn relevant features directly. However, this also means that it is not straightforward to incorporate prior information into the models. For example, the binding preferences for many RNA- and DNA-binding proteins are already known and cataloged [38,39]. To take advantage of this information, the authors of OrbWeaver [40] fixed the first layer convolutional filters to 1320 known transcription factor motifs and found that on their small dataset of three cell types, this configuration outperformed a classical network that tried to learn motifs from the data. Furthermore, the fixed motifs were easier to interpret with DeepLIFT [41]. Similarly, the authors of DanQ [34] increased the accuracy of the model by initializing 50% of the convolutional filters in the first layer with known transcription factor motifs, but allowing them to change during training.

## Biological image analysis

As some of the most impressive feats of deep neural networks have been in image analysis tasks, the expectations are high for their utility in bioimage analyses. Microscopy images are processed with manufacturer's software (e.g. PerkinElmer Acapella) or community-driven tools such as CellProfiler [42], EBImage [43], or Fiji [44] that have evolved to user demands over many years. What capabilities have neural networks recently added to this rich existing toolbox?

### Image segmentation

Segmentation identifies regions of interest, such as cells or nuclei, within a microscopy image, a task equivalent to classifying each pixel as being inside or outside of the region. The early neural network applications trained a convolutional network on square image patches centered on labeled pixels [45] and performed well in open challenges [46]. Recently, Van Valen et al. adopted this approach in a high-content screening setting and used it to segment both mammalian and bacterial cells [47]. Perhaps most importantly, they identified the optimal input size to the neural network to be similar to the typical size of the region of interest.

An alternative to classifying the focal pixel within its surrounding region is to perform end-to-end image segmentation. U-net [48] achieved this with a fully convolutional design, where image patch features are calculated at a range of resolutions by convolution and pooling, and then combined across the resolutions to produce a prediction for each pixel. The architecture of the network, therefore, included links that feed the early layer outputs forward to deeper layers in order to retain the localization information. Segmentation approaches have since been extended to handle 3D images by applying U-net to 2D slices from the same volume [49], and by performing 3D convolutions [50].

Recent applications of deep neural networks to segment medical imaging data have been thoroughly reviewed elsewhere [51–53]; we cover some histopathology studies in the Appendix to this article.

### Cell and image phenotyping

Segmenting regions of interest is the starting point of biological image analysis. One desired end product is a cell phenotype, which captures cell state either qualitatively or quantitatively [54]. Previous methods for obtaining phenotypes have ranged from low-level image processing transforms that can be applied to any image (Gabor or Zernicke filters, Haralick features, a range of signal processing tools, [55]), to bespoke crafting of features that precisely capture the desired image characteristic in a given dataset [56,57] and unsupervised clustering of full images [58]. An important intermediate approach is to learn informative features from a given dataset *de novo*, a task that deep neural networks excel at.

A recurring phenotyping problem is to identify the subcellular localization of a fluorescent protein. Pärnamaa and Parts used convolutional neural networks with a popular design (e.g. also applied for plant phenotyping, [59]) to solve this task with high accuracy for images of single yeast cells [60] obtained in a high-content screen [56]. They employed eight convolutional layers of 3 × 3 filters interspersed with pooling steps, which were followed by three fully connected layers that learn the feature combinations that discriminate organelles. The learned features were interpretable, capturing organelle characteristics, and robust, allowing us to predict previously unseen organelles after training on a few examples. The authors further combined cell-level predictions into a single, highly accurate, protein classification. A team from Toronto demonstrated on the same unsegmented data that are possible to identify a localization label within a region and an image-level label with convolutional neural networks in a single step [61]. This has the advantage that only image-level labels are used, precluding the need to perform cell segmentation first. The output of the model, thus, also provides a per-pixel localization probability that could further be processed to perform segmentation.

Much of the recent effort has been in obtaining qualitative descriptions of individual cells. Convolutional neural networks could accurately detect phototoxicity [62] and cell-cycle states [63] from images. An interesting architecture predicts lineage choice from brightfield timecourse imaging of differentiating primary hematopoietic progenitors by combining convolution for individual micrographs with recurrent connections between timepoints [64]. Markedly, the lineage commitment can be predicted up to three generations before conventional molecular markers are observed.

Instead of a discrete label, a vector of quantitative features describing the cell or image can be useful in downstream applications. One approach to calculate this representation is to re-use a network trained on colossal datasets as a feature extractor. For example, cellular microscopy images can be phenotyped using the features obtained from such pre-trained networks [65]. Alternatively, autoencoders (Box 1) attempt to reconstruct the input by a neural network with a limited number of neurons in one of the layers, similar to an independent component analysis model. Neuron activations in the smallest layer can then be used as features for other machine learning methods; importantly, these are learned from data each time. This approach has been used to aid diagnoses for schizophrenia [66], brain tumors [67], lesions in the breast tissue [68,69], and atherosclerosis [70].

## Medical diagnostics

The ultimate goal of much of biomedical research is to help diagnose, treat, and monitor patients. The popularity of deep learning has, thus, naturally led to public–private partnerships in diagnostics, with IBM's Watson tackling cancer and Google's DeepMind Health teaming up with the National Health Service in the U.K. While the models are being industrialized, many interesting advances in applications occurred over the last year.

### Self-diagnosis with deep learning

Neural networks have become universally available through mobile applications and web services. Provided useful pre-trained models, this could allow everyone to self-diagnose on their phone and only refer to the hospital for the required treatments. As a first step toward this vision, the GoogLeNet convolutional neural network [71] was re-trained on ∼130 000 images of skin lesions, each labeled with a malignancy indicator from a predefined taxonomy [72]. The classification performance on held-out data was on par with that of professionally trained dermatologists. Thus, this network could be capable of instantly analyzing and diagnosing birthmark images taken from regular smartphones, allowing us to detect skin cancer cases earlier and hence increase survival rates.

The problem, however, is that any one image with a malignant lesion could be marked as benign. A natural resolution to this issue is to further endow the convolutional neural network with an uncertainty estimate of its output [73]. This estimate is obtained by applying the model on the same image many times over, but with a different set of random neurons switched off each time (‘dropout’, [74]). The larger the changes in output in response to the randomization, the higher the model uncertainty, and importantly, the larger the observed prediction error. Images with large classification uncertainty could then be sent to human experts for further inspection, or simply re-photographed.

More than images can be captured using a phone. Chamberlain et al. [75] recorded 11 627 lung sounds from 284 patients using a mobile phone application and an electronic stethoscope, and trained an autoencoder (Box 1) to learn a useful representation of the data. Using the extracted features, and 890 labels obtained via a laborious process, two support vector machine classifiers were trained to accurately recognize wheezes and crackles, important clinical markers of pulmonary disease. As a stand-alone mobile application, these models could help doctors from around the world to recognize signs of the disease. In a similar vein, deep neural networks have been applied to diagnose Parkinson disease from voice recordings [76] and to classify infant cries into ‘hunger’, ‘sleep’, and ‘pain’ classes [77].

Other clinical assays that are relatively easy to perform independently could be analyzed automatically. For example, the heart rate and QT interval of 15 children with type 1 diabetes were monitored overnight and used to accurately predict low blood glucose with a deep neural network model [78]. Aging.ai, which uses an ensemble of deep neural networks on 41 standardized blood test measurements, has been trained to predict an individual's chronological age [79].

### Using other medical data modalities

Computer tomography (CT) is a precise, but costly and risky procedure, while magnetic resonance imaging (MRI) is safer, but noisier. Nie et al*.* [80] trained a model to generate CT scan images from MRI data. To do so, they employed a two-part model, where one convolutional neural network was trained to generate CT images from MRI information, while the other was trained to distinguish between true and generated ones. As a result, the MRI images could be converted to CT scans that qualitatively and quantitatively resembled the true versions. This is the first application of generative adversarial networks (Box 1) [81], a recently popularized method, for medical data.

Electronic health records are a prime target for medical data models. In Doctor AI, past diagnoses, medication, and procedure codes were inputted to a recurrent neural network to predict diagnoses and medication categories for subsequent visits, beating several baselines [82]. Three layers of autoencoders were used to capture hierarchical dependencies in aggregated electronic health records of 700 000 patients from the Mount Sinai data warehouse [83]. This gave a quantitative latent description of patients which improved classification accuracy, and provided a compact data representation.

A range of other medical input signals has been usefully modeled with neural networks. Al Rahhal et al. [84] trained autoencoders to learn features from electrocardiogram signals and used them to detect various heart-related disorders. As a completely different input, a video recording of a patient's face could be used to automatically estimate pain intensity with a recurrent convolutional neural network [85]. Just over the last year, there have been reports of applying convolutional neural networks in image-based diagnostics of age-related macular degeneration [86], diabetic retinopathy [87], breast cancer [88–90], brain tumors [91,92], cardiovascular disease [93], Alzheimer's disease [94], and many more diseases (Appendix to this article).

## Discussion

Deep learning has already permeated computational biology research. Yet its models remain opaque, as the inner workings of the deep networks are difficult to interpret. The layers of convolutional neural networks can be visualized in various ways to understand input features they capture, either by finding real inputs that maximize the neuron outputs, e.g. [60], generating synthetic inputs that maximize the neuron output [95], or mapping inputs that the neuron output is most sensitive to (saliency map, [96]; or alternative [97]). In this manner, neurons operating on sequences could be interpreted as detecting motifs and their combinations, or neurons in image analysis networks as pattern finders. All these descriptions are necessarily qualitative, so conclusive causal claims about network performance due to capturing a particular type of signal are to be taken with a grain of salt.

Computer performance in image recognition has reached human levels, owing to the volume of available high-quality training datasets [98]. The same scale of labeled biological data is usually not obtainable, so deep learning models trained on a single new experiment are bound to suffer from overfitting. However, one can use networks pre-trained on larger datasets in another domain to solve the problem in hand. This transfer learning can be used both as a means to extract features known to be informative in other applications and as a starting point for model fine-tuning. Repositories of pre-trained models are already emerging (e.g. Caffe Model Zoo) and first examples of transfer learning have been successful [72,99], so we expect many more projects to make use of this idea in the near future.

Will deep learning make all other models obsolete? Neural networks harbor hundreds of parameters to be learned from the data. Even if sufficient training data exist to make a model that can reliably estimate them, the issues with interpretability and generalization to data gathered in other laboratories under other conditions remain. While deep learning can produce exquisitely accurate predictors, the ultimate goal of research is understanding, which requires a mechanistic model of the world.

## Summary

- Deep learning methods have penetrated computational biology research.
- Their applications have been fruitful across functional genomics, image analysis, and medical informatics.
- While trendy at the moment, they will eventually take a place in a list of possible tools to apply, and complement, not supplement, existing approaches.

## Appendix

**Table:** Short overview of computational biology deep learning papers published until the first quarter of 2017

Name	Title	Architecture	Input	Output	Highlight	Category
**FUNCTIONAL GENOMICS**						
DeepBind	Predicting the sequence specificities of DNA- and RNA-binding proteins by deep learning [5]	CNN	DNA sequence	TF binding	Arbitrary length sequences	DNA binding
DeeperBind	DeeperBind: enhancing prediction of sequence specificities of DNA binding proteins [35]	CNN-RNN	DNA sequence	TF binding	Sequences of arbitrary length. Adds LSTM to DeepBind model.	DNA binding
DeepSEA	Predicting effects of noncoding variants with deep learning-based sequence model [7]	CNN	DNA sequence	TF binding	3-layer CNN	DNA binding
DanQ	DanQ: a hybrid convolutional and recurrent deep neural network for quantifying the function of DNA sequences [34]	CNN-RNN	DNA sequence	TF binding	Adds LSTM layer to DeepSEA model	DNA binding
TFImpute	Imputation for transcription factor binding predictions based on deep learning [14]	CNN	DNA sequence; ChIP-seq	TF binding	Impute TF binding in unmeasured cell types	DNA binding
Basset	Basset: learning the regulatory code of the accessible genome with deep convolutional neural networks [6]	CNN	DNA sequence	Chromatin accessibility	Uses DNAse-seq data from 164 cell types	DNA binding
OrbWeaver	Impact of regulatory variation across human iPSCs and differentiated cells [40]	CNN	DNA sequence	Chromatin accessibility	Uses known TF motifs as fixed filters in the CNN	DNA binding
CODA	Denoising genome-wide histone ChIP-seq with convolutional neural networks [15]	CNN	ChIP-seq	ChIP-seq	Denoise ChiP-seq data	DNA binding
DeepEnhancer	DeepEnhancer: predicting enhancers by convolutional neural networks [10]	CNN	DNA sequence	Enhancer prediction	Convert convolutional filters to PWMs, compare to motif databases	DNA binding
TIDE	TIDE: predicting translation initiation sites by deep learning [12]	CNN-RNN	RNA sequence	Translation initiation sites (QTI-seq)	DanQ model	RNA binding
ROSE	ROSE: a deep learning based framework for predicting ribosome stalling [101]	CNN	RNA sequence	Ribosome stalling (ribosome profiling)	Parallel convolutions	RNA binding
iDeep	RNA-protein binding motifs mining with a new hybrid deep learning based cross-domain knowledge integration approach [21]	CNN-DBN	RNA sequence;	RNA binding proteins (CLiP-seq)	Integrate multiple diverse data sources	RNA binding
			Known motifs			
			Secondary structure			
			co-binding			
			transcript region			
Deepnet-rbp	A deep learning framework for modeling structural features of RNA-binding protein targets [20]	DBN	RNA sequence	RNA binding proteins (CLiP-seq)	Uses k-mer counts instead of a CNN to capture RNA sequence features	RNA binding
			secondary structure			
			tertiary structure			
SPEID	Predicting enhancer-promoter interaction from genomic sequence with deep neural networks [13]	CNN-RNN	DNA sequence	Promoter-enhancer interactions	Inspired by DanQ	3D interactions
Rambutan	Nucleotide sequence and DNaseI sensitivity are predictive of 3D chromatin architecture [18]	CNN	DNA sequence	Hi-C interactions	Binarised input signal	3D interactions
			DNAse-seq			
			Genomic distance			
DeepChrome	A deep learning framework for modeling structural features of RNA-binding protein targets [20]	CNN	Histone modification (ChIP-seq)	Gene expression	Binary decision: expressed or not expressed	Transcription
FIDDLE	FIDDLE: An integrative deep learning framework for functional genomic data inference [16]	CNN	DNA sequence	Transcription start sites (TSS-seq)	DNA sequences alone not sufficient for prediction, other data helps	Transcription
			RNA-seq			
			NET-seq			
			MNAse-seq			
			ChIP-seq			
CNNProm	Recognition of prokaryotic and eukaryotic promoters using convolutional deep learning neural networks [9]	CNN	DNA sequence	Promoter predictions	Predicts promoters from DNA sequnce features	Transcription
DeepCpG	DeepCpG: accurate prediction of single-cell DNA methylation states using deep learning [19]	CNN-GRU	DNA sequence	DNA methylation state (binary)	Predict DNA methylation state in single cells based on sequence content (CNN) and noisy measurement (GRU)	DNA methylation
			scRRBS-seq			
CpGenie	Predicting the impact of non-coding variants on DNA methylation [11]	CNN	DNA sequence	DNA methylation state (binary)	Predict genetic variants that regulate DNA methyaltion	DNA methylation
DNN-HMM	De novo identification of replication-timing domains in the human genome by deep learning [102]	Hidden markov model (HMM) combinded with deep belief network (DBN)	Replicated DNA sequencing (Repli-seq)	Replication timing	Predict replication timing domains from Repli-seq data	Other
DeepCons	Understanding sequence conservation with deep learning [8]	CNN	DNA sequence	Sequence conservation	Works on noncoding sequences only	Other
GMFR-CNN	GMFR-CNN: an integration of gapped motif feature representation and deep learning approach for enhancer prediction [103]	CNN	DNA sequence	TF binding	Uses data from the DeepBind paper. Integrates gapped DNA motifs (as introduced by gkm-SVM) with a convolutional neural network	DNA binding
**SEQUENCE DATA ANALYSIS**						
DeepVariant	Creating a universal SNP and small indel variant caller with deep neural networks [32]	CNN	Image	Assignment of low confidence variant call (Illumina sequencing)	Turns sequence, base quality, and strand information into image	Basecalling
Goby	Compression of structured high-throughput sequencing data [104]	Dense	Features	Base call (Illumina sequencing)	Part of wider variant calling framework	Basecalling
DeepNano	DeepNano: Deep Recurrent Neural Networks for Base Calling in MinION Nanopore Reads [33]	RNN	Current	Base call (nanopore sequencing)	Uses raw nanopore sequencing signal	Basecalling
-	Deep learning for population genetic inference [25]	Dense	Features	Effective population size; selection coefficient	Estimate multiple population genetic parameters in one model	Population genetics
**MEDICAL DIAGNOSTICS**						
	Leveraging uncertainty information from deep neural networks for disease detection [73]	BCNN	Image (retina)	Disease probability	For each image estimates an uncertainty of the network, if this uncertainty is too high, discards image	Medical diagnostics
DRIU	Deep retinal image understanding [105]	CNN	Image (retina)	Segmentation	Super-human performance, task customised layers	Retinal segmentation
IDx-DR X2.1	Improved automated detection of diabetic retinopathy on a publicly available dataset through integration of deep learning [87]	CNN	Image (retina)	DR stages	Added DL component into the algorithm and reported its superior performance	DR detection
	Deep learning is effective for classifying normal versus age-related macular degeneration OCT images [86]	CNN (VGG16)	Image (OCT)	Normal versus Age-related macular degeneration	Visualised salience maps to confirm that areas of high interest for the network match pathology areas	Age-related macular degeneration classification
	Medical image synthesis with context-aware generative adversarial networks [80]	GAN	Image (MR patch)	CT patch	Predicts CT image from 3D MRI, could also be used for super-resolution, image denoising etc	Medical image synthesis
DeepAD	DeepAD: Alzheimer's disease classification via deep convolutional neural networks using MRI and fMRI [94]	CNN	Image (fMRI and MRI)	AD vs NC	99.9% accuracy for LeNet architecture, fishy	Alzheimer's disease classification
	Brain tumor segmentation with deep neural networks [91]	CNN	Image (MRI)	Segmentation of the brain	Stacked CNNs, fast implementation	Glioblastoma
	Brain tumor segmentation using convolutional neural networks in MRI images [92]	CNN	Image (MRI)	Segmentation of the brain		
	A deep learning-based segmentation method for brain tumor in MR images [67]	SDAE + DNN	Image (MRI)	Segmentation of the brain		
	Classification of schizophrenia versus normal subjects using deep learning [66]	SAE + SVM	Image (3D fMRI volume)	Disease probability	Works on directly on active voxel time series without conversion	Schizophrenia classification
	Predicting brain age with deep learning from raw imaging data results in a reliable and heritable biomarker [106]	3D CNN	Image (minimally preprocessed raw T1-weighted MRI data)	Age	Almost no preprocessing, brain age was shown to be heritable	Age prediction
	Mass detection in digital breast tomosynthesis: deep convolutional neural network with transfer learning from mammography [107]	CNN	Image (mammography + DBT)	Disease probability	Network was first trained on mammography images, then first three conv. layers were fixed while other layers were initialised and trained again on DBT (Transfer Learning)	Medical diagnostics + Transfer Learning
	Large scale deep learning for computer aided detection of mammographic lesions [90]	CNN + RF	Image (mammography patch)	Disease probability	Combines handcrafted features with learned by CNN to train RF	Mammography lesions classification
DeepMammo	Breast mass classification from mammograms using deep convolutional neural networks [89]	CNN	Image (mammography patch)	Disease probability	Transfer learning from pre-trained CNNs	Mammography lesions classification
	Unsupervised deep learning applied to breast density segmentation and mammographic risk scoring [68]	CSAE	Image (mammogram)	Segmentation and classification of lesions	Developed a novel regularisor	Mammography segmentation and classification
	A deep learning approach for the analysis of masses in mammograms with minimal user intervention [88]	CNN + DBN	Image (mammogram)	Benign vs malignant class	End to end approach with minimal user intervention, some small tech innovation at each stage	Mammography segmentation and classification
	Detecting cardiovascular disease from mammograms with deep learning [93]	CNN	Image (mammogram patch)	BAC vs normal	Using mammograms for cardiovascular disease diagnosis	Breast arterial calcifications detection
	Lung pattern classification for interstitial lung disease using a deep convolutional neural network [108]	CNN	Image (CT patch)	7 ILD classes	Maybe the first attempt to characterize lung tissue with deep CNN tailored for the problem	Medical diagnostics
	Multi-source transfer learning with convolutional neural networks for lung pattern analysis [109]	CNN	Image (CT patch)	7 ILD classes	Transfer learning + ensemble	
	Deep convolutional neural networks for computer-aided detection: CNN architectures, dataset characteristics and transfer learning [110]	CNN	Image (CT)	ILD classes and Lung Node detection	Transfer learning, many architectures, IDL and LN detection	
	Computer-aided diagnosis with deep learning architecture: applications to breast lesions in us images and pulmonary nodules in CT scans [69]	SDAE	Image (US and CT ROI)	Benign vs malignant class	Used the same SDAE for both breast lesions in US images and pulmonary nodules in CT scans, concatenated handcrafted features to original ROI pixels	CAD
	Dermatologist-level classification of skin cancer with deep neural networks [72]	CNN	Image (Skin)	Disease classes	Could be potentially used on a server side to power self-diagnosis of skin cancer	Medical diagnostics
	Early-stage atherosclerosis detection using deep learning over carotid ultrasound images [70]	AE	Image (US)	Segmentation and classification of arterial layers	Fully automatic US segmentation	Intima-media thickness measurement
	Fusing deep learned and hand-crafted features of appearance, shape, and dynamics for automatic pain estimation [111]	CNN + LR	Image (Face)	Pain intensity	Combines handcrafted features with learned by CNN to train Linear regressor	Pain intensity estimation
	Recurrent convolutional neural network regression for continuous pain intensity estimation in video [85]	RCNN	Video frames	Pain intensity		Pain intensity estimation
	Efficient diagnosis system for Parkinson's disease using deep belief network [76]	DBN	Sound (Speech)	Parkinson vs normal		Parkinson diagnosis
	Application of semi-supervised deep learning to lung sound analysis [75]	DA + 2 SVM	Sound (Lung sounds)	Sound scores	Handling small data sets with DA + potential application	Pulmonary disease diagnosis
	Application of deep learning for recognizing infant cries [77]	CNN	Sound (Infant cry)	Class scores		Sound classification
	Deep learning framework for detection of hypoglycemic episodes in children with type 1 diabetes [78]	DBN	ECG	Hypoglycemic episode onset	Real-time episodes detection	Hypoglycemic episodes detection
	Deep learning approach for active classification of electrocardiogram signals [84]	SDAE	ECG	AAMI classes	Uses raw ECG	Classification of electrocardiogram signals
AgingAI	Deep biomarkers of human aging: application of deep neural networks to biomarker development [79]	21 DNN	Blood test measurements	Age	Online tool which could be used to collect training data, 5 biomarkers for aging	Age prediction
**BIOMEDICAL IMAGE ANALYSIS**						
**Image segmentation**						
DeepCell	Deep learning automates the quantitative analysis of individual cells in live-cell imaging experiments [47]	CNN	Microscopy images	Cell segmentations	Able to segment both mammalian and bacterial cells	Segmentation
U-Net	U-Net: convolutional networks for biomedical image segmentation [48]	CNN	Biomedical images	Segmentations	Won the ISBI 2015 EM segmentation challenge	Segmentation
3D U-Net	3D U-Net: learning dense volumetric segmentation from sparse annotation [49]	CNN	Volumetic images	3D Segmentations	Able to quickly volumetric images	Segmentation
V-Net	V-Net: Fully convolutional neural networks for volumetric medical image segmentation [50]	CNN	Volumetic images	3D Segmentations	Performs 3D convolutions	Segmentation
**Cell and image phenotyping**						
DeepYeast	Accurate classification of protein subcellular localization from high throughput microscopy images using deep learning [60]	CNN	Microscopy images	Yeast protein localisation classification		Automatic Phenotyping
	Deep machine learning provides state-of-the-art performance in image-based plant phenotyping [59]	CNN	Plant images	Plant section phenotyping		Automatic Phenotyping
	Classifying and segmenting microscopy images with deep multiple instance learning [61]	CNN	Microscopy images	Yeast protein localisation classification	Performs multi-instance localisation	Automatic Phenotyping
DeadNet	DeadNet: identifying phototoxicity from label-free microscopy images of cells using Deep ConvNets [62]	CNN	Microscopy images	Phototoxicity identification		Automatic Phenotyping
	Deep learning for imaging flow cytometry: cell cycle analysis of Jurkat cells [63]	CNN	Single cell microscopy images	Cell-cycle prediction		Automatic Phenotyping
	Prospective identification of hematopoietic lineage choice by deep learning [64]	CNN	Brightfield time course imaging	Hematopoitic lineage choice	Lineage choice can be detected up to three generations before conventional molecular markers are observable	Automatic Phenotyping
	Automating morphological profiling with generic deep convolutional networks [65]	CNN	Microscopy images	Feature extraction		Automatic Phenotyping

While we have tried to be comprehensive, some papers may have been missed due to the rapid development of the field. Acronyms used: AE, autoencoder; BCNN, bayesian convolutional neural network; CNN, convolutional neural network; CSAE, convolutional sparse autoencoder; DA, denoising autoencoder; DBN, deep belief network; GAN, generative adversarial network; GRU, gated recurrent unit; LR, linear regression; RCNN, recurrent convolutional neural network; RF, random forest; RNN, recurrent neural network; SAE, stacked autoencoder; SDAE, stacked denoising auto-encoder; SVM, support vector machines.

## Abbreviations

CT: computer tomography
MRI: magnetic resonance imaging
